# Physics-driven proper orthogonal decomposition: A simulation methodology for partial differential equations

**DOI:** 10.1016/j.mex.2023.102204

**Published:** 2023-04-28

**Authors:** Alessandro Pulimeno, Graham Coates-Farley, Martin Veresko, Lin Jiang, Ming-Cheng Cheng, Yu Liu, Daqing Hou

**Affiliations:** a Department of Electrical and Computing Engineering, Clarkson University, Potsdam, NY 13699, USA; b Department of Mechanical and Aerospace Engineering, Clarkson University, Potsdam, NY 13699, USA; c Current affiliation, Department of Aerospace Engineering, Delft University of Technology, Delft, The Netherlands

**Keywords:** Heat transfer, Quantum nanostructures, Machine learning, Partial differential equations, Galerkin projection, Physics-driven simulation methodology based on Proper Orthogonal Decomposition enabled by Galerkin projection (POD-GP)

## Abstract

A simulation methodology derived from a learning algorithm based on Proper Orthogonal Decomposition (POD) is presented to solve partial differential equations (PDEs) for physical problems of interest. Using the developed methodology, a physical problem of interest is projected onto a functional space described by a set of basis functions (or POD modes) that are trained via the POD by solution data collected from direct numerical simulations (DNSs) of the PDE. The Galerkin projection of the PDE is then performed to account for physical principles guided by the PDE. The procedure to construct the physics-driven POD-Galerkin simulation methodology is presented in detail, together with demonstrations of POD-Galerkin simulations of dynamic thermal analysis on a microprocessor and the Schrödinger equation for a quantum nanostructure. The physics-driven methodology allows a reduction of several orders in degrees of freedom (DoF) while maintaining high accuracy. This leads to a drastic decrease in computational effort when compared with DNS. The major steps for implementing the methodology include:

• Solution data collection from DNSs of the physical problem subjected to parametric variations of the system.
• Calculations of POD modes and eigenvalues from the collected data using the method of snapshots.
• Galerkin projection of the governing equation onto the POD space to derive the model.

## Graphical abstract

**Figure:**
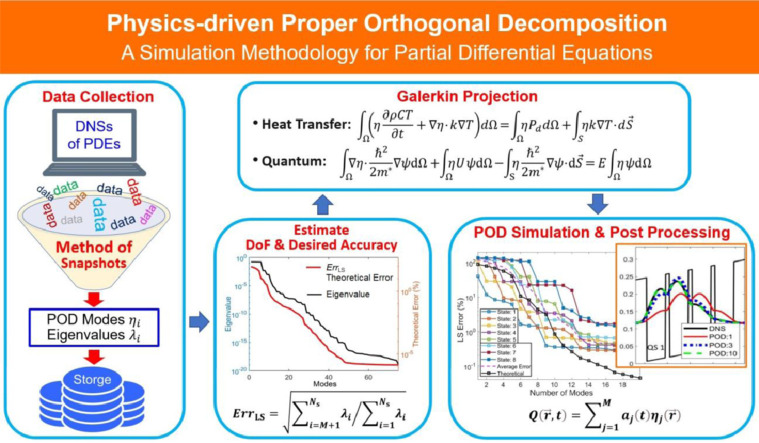

Specifications table

**Table:** 

Subject area:	Engineering
More specific subject area:	Data-driven engineering and science, Physical simulations
Name of your method:	Physics-driven simulation methodology based on Proper Orthogonal Decomposition enabled by Galerkin projection (POD-GP)
Name and reference of original method:	Lin Jiang, Martin Veresko, Yu Liu, and Ming-C. Cheng. 2022. An effective physics simulation methodology based on a data-driven learning algorithm. In Proceedings of the Platform for Advanced Scientific Computing Conference (PASC '22). Association for Computing Machinery, New York, NY, USA, Article 16, 1...10. https://doi.org/10.1145/3539781.3539799
Resource availability:	https://github.com/CompResearchLab/

## Background

A significant portion of scientific and engineering problems relies on Partial Differential Equations (PDEs) whose solution is often obtained from direct numerical simulation (DNS) based on the finite difference method (FDM), finite element method (FEM) or finite volume method (FVM). The drawback to DNS methods is the immense amount of computational time and memory space required in the simulations, especially in large simulation domains with high resolution [1], [2], [3]. In recent years, engineering and scientific problems are growing more complex due to the demands for more accurate solutions and more sophisticated functions. To satisfy the growing demands, more sophisticated, accurate DNSs for the problem of interest are needed, which results in even more computational time and memory space. This presented a severe issue for researchers and engineers to obtain simulation results in an acceptable effort for analysis/design of systems of their interest. To resolve this obstacle, the simulation methodology of data-driven Proper Orthogonal Decomposition (POD) enabled by Galerkin projection was developed as the solution [4].

The POD simulation method was first introduced in the context of turbulence by Lumley in 1967 [5]. In his works, Lumley shows that the simplicity and robustness of POD is hidden in the fact that it is a linear procedure [5,6]. The POD is a method that extracts an optimal set of basis functions (hereafter referred to as POD modes) for a modal decomposition from a set of measurements or numerical data [4], [5], [6]. This procedure, together with the Galerkin projection, converts the problem into a low-dimensional POD functional space described by these basis functions. As a consequence, this physics-driven POD-Galerkin learning methodology offers a reduction in Degrees of Freedom (DoF) by several orders of magnitude with a significant degree of accuracy [3,4,7,8].

To respond to the demand for accelerating simulation for complex systems in recent years, more efficient methods need to be developed. In this tutorial, the POD-Galerkin methodology, a data-driven learning technique, is presented to profoundly reduce the DoF, and hence the computational time and effort, without affecting the desired accuracy of the results. However, the accuracy of the solution highly depends on the quality of data collected in the training process [3,4]. After generating the POD modes using the good-quality data collected from DNSs, the Galerkin projection method is used to project the governing equation of the problem onto a functional space represented by these POD modes. This is a crucial step that enforces the POD simulation to comply with the physical principles offered by the governing equation [3], which reinforces its high accuracy using a small number of DoF with the extrapolation capability. This is a key difference from most machine learning methods based only on learning from data, where statistical approaches are applied to minimize the variation of the prediction without considering physical principles. These machine learning methods, unlike the POD-Galerkin methodology, may lead to a prediction inconsistent with the underline physical principles in case of extrapolation. Many recent studies have successfully incorporated physical principles into various deep learning-based methodologies [9], [10], [11], [12] and have shown improved performance over the purely learning-based methodologies, especially in cases of extrapolations. However, these physis-informed deep learning methods do not provide schemes to predict the error. On the contrary, the LS error for the physis-drive POD-Galerkin methodology can be reasonably predicted from the POD eigenvalues, as will be demonstrated later in this work.

With an optimal set of basis functions and crucial physical principles incorporated in the POD-Galerkin simulation methodology, the approach is able to achieve high accuracy, great efficiency, and fine resolution with no priori assumptions for problems governed by PDEs. The step-by-step procedure and concepts of the POD-Galerkin simulation methodology are presented in this work to illustrate its physics-driven learning ability for a heat transfer problem on a microprocessor and a quantum eigenvalue problem of a nanostructure. The former can be applied to effective predictions of dynamic thermal profiles on multi-core processors for reliability assessment [13,14] as well as for thermal-aware real-time management to suppress hot-spot formation [14,15]. The latter may be extended to many scientific and engineering problems that require eigen solutions, including acoustic and electromagnetic metamaterials [16], [17], [18]. Although very efficient and accurate, the major drawback of the POD-Galerkin methodology is the computationally intensive training process needed for large-scale multi-dimensional structures [3,4], which could become computationally prohibitive for a large-scale structure with fine resolution. To effectively minimize the training effort, domain decomposition can be incorporated in the methodology, which partitions the large-scale structure into smaller blocks whose POD training is relatively efficient with small memory space, which will be investigated in the near future.

## Data collection

As stated above, the accuracy of the POD solutions depends strongly on the quality of the data obtained from DNSs for the training of the POD modes. The data quality is twofold. First, the data ensemble needs to cover a broad range of parametric variations for the trained POD modes to encompass a range of possible scenarios. This thus depends on the number of parameters that need to vary to account for possible combinations of operating parameters in simulation of the system. A large number of combinations may impose an intensive training effort. Second, accuracy of the data collected from DNSs is crucial to ensure the quality of the generated POD modes and thus the accurate prediction of the POD-Galerkin simulation. This is particularly important in regions with high gradient variation, where a finer mesh is needed to ensure accurate numerical gradients to be able to generate a set of good-quality modes. The solution data for the training can be obtained from any commercial or open-source DNS tools that offer accurate numerical solutions.

### Proper orthogonal decomposition

Once sufficient data are collected to account for parametric variations over the entire domain of interest, each of the basis functions (POD modes) $\eta (\vec{r})$ is generated by maximizing the mean square of the inner product between the solution $Q(\vec{r},t)$ and the mode $\eta (\vec{r})$ [5,6]:

(1) $$\frac{\langle {\left(\int_{\Omega }Q\left(\vec{r},t\right)\eta \left(\vec{r}\right)d\Omega \right)}^{2}\rangle }{\int_{\Omega }\eta {\left(\vec{r}\right)}^{2}d\Omega },$$

where Ω represents the selected physical domain, and the angle brackets 〈〉 indicate the average over multiple samples of numerical observations that account for parametric variations. Note that for a transient problem, the solution data from DNSs are sampled at different instants in time to account for parametric variations. For a static problem, they are sampled to collect solution data in response to variations of internal and external disturbances/excitations. For example, these disturbances or excitations may include boundary conditions (BCs) and heat excitations for a heat transfer problem or potential variations in a nanostructure. This maximization process ensures that the generated mode $\eta (\vec{r})$ offers the best least squares (LS) fit to the solution of the problem.

The maximization procedure is repeated to generate the next mode orthogonal to the previous one. In this manner, an orthogonal set of POD modes can be derived. Applying variational calculus to the maximization problem given in Eq. (1), an eigenvalue problem given by the Fredholm equation is found [5,6]:

(2) $$\int_{\Omega^{\prime }}R\left(\vec{r},{\vec{r}}^{{}^{\prime }}\right)\eta \left({\vec{r}}^{{}^{\prime }}\right)d\Omega^{\prime }=\lambda \eta \left(\vec{r}\right),$$

Where *λ* is the POD eigenvalue of the two-point correlation matrix $R(\vec{r},{\vec{r}}^{{}^{\prime }})$. *λ* represents the mean squared variation of Q captured by its POD mode, which reveals the importance of the corresponding mode in the LS sense. The two-point correlation matrix is expressed as:

(3) $$R\left(\vec{r},{\vec{r}}^{{}^{\prime }}\right)=\;\langle Q\left(\vec{r},t\right)⊗Q\left({\vec{r}}^{{}^{\prime }},t\right)\rangle ,$$

where ⊗ is the tensor product.

For discrete data in space, Eq. (2) represents an eigenvalue problem with a dimension of $N_{r}\times N_{r}$, where $N_{r}$ is the number of grid points in the domain. In a large multi-dimensional domain, a large amount of computational resources (both storage and computational time) is needed to solve the eigenvalue problem. Instead of solving Eq. (2) directly for the POD modes and eigenvalues, the method of snapshots [19,20] is applied, which converts the problem from an $N_{r}\times N_{r}$ domain to an $N_{s}\times N_{s}$ eigenvalue problem, where $N_{s}$ is the number of samples (i.e. snapshots) and $N_{s}\ll N_{r}$. This reduces considerably the dimensionality of the problem.

### Method of snapshots for calculations of pod modes

From Eq. (2), the average of the tensor product can be expressed as follows [19,20]:

(4) $$\langle Q\left(\vec{r},t\right)⊗Q\left({\vec{r}}^{{}^{\prime }},t\right)\rangle =\frac{1}{N_{s}}\sum_{j=1}^{N_{s}}Q\left(\vec{r},t_{j}\right)Q\left({\vec{r}}^{{}^{\prime }},t_{j}\right).$$

Hence, the eigenvalue problem in Eq. (2) can be rewritten as:

(5) $$\frac{1}{N_{s}}\sum_{j=1}^{N_{S}}Q\left(\vec{r},t_{j}\right)\int_{\Omega^{\prime }}Q\left({\vec{r}}^{{}^{\prime }},t_{j}\right)\eta \left({\vec{r}}^{{}^{\prime }}\right)d\Omega^{\prime }=\lambda \eta \left(\vec{r}\right).$$

Now, define the projection of the sampled data onto the POD space as:

(6) $$u\left(t_{j}\right)=\int_{\Omega^{\prime }}Q\left({\vec{r}}^{{}^{\prime }},t_{j}\right)\eta \left({\vec{r}}^{{}^{\prime }}\right)d\Omega^{\prime },$$

and the eigenvalue problem of Eq. (5) then becomes:

(7) $$\frac{1}{N_{s}}\sum_{j=1}^{N_{S}}Q\left(\vec{r},t_{j}\right)u\left(t_{j}\right)=\lambda \eta \left(\vec{r}\right).$$

Multiplying both sides of Eq. (7) by $Q(\vec{r},t_{i})\;$and performing integration over the domain Ω, the following equation is derived:

(8) $$\frac{1}{N_{s}}\sum_{j=1}^{N_{s}}\left[\int_{\Omega }Q\left(\vec{r},t_{i}\right)Q\left(\vec{r},t_{j}\right)d\Omega \right]u\left(t_{j}\right)=\lambda \int_{\Omega }Q\left(\vec{r},t_{i}\right)\eta \left(\vec{r}\right)d\Omega .$$

With the matrix element defined as:

(9) $$A_{ij}=\frac{1}{N_{s}}\int_{\Omega }Q\left(\vec{r},t_{i}\right)Q\left(\vec{r},t_{j}\right)d\Omega ,$$

the eigenvalue problem in the sampling domain can be written as:

(10) $$A\vec{u}=\lambda \vec{u}.$$

Once the eigenvectors are determined, the POD modes can be obtained via the linear combination of numerical observations:

(11) $$\eta_{i}\left(\vec{r}\right)=\frac{1}{\lambda N_{s}}\sum_{j=1}^{N_{s}}Q\left(\vec{r},t_{j}\right)u_{i}\left(t_{j}\right).$$

### Accuracy metrics

Once the POD modes are found, the solution $Q(\vec{r},t)$ can be expressed as a linear combination of the modes:

(12) $$Q\left(\vec{r},t\right)=\sum_{j=1}^{M}a_{j}\left(t\right)\eta_{j}\left(\vec{r}\right),$$

Where M is the selected number of modes (or DoF) to represent the solution, $M\leq N_{s}$, and $a_{j}$ are the weighting coefficients. To close the system, the Galerkin projection is applied to derive a set of equations for $a_{j}$, which is presented in the following sections for the selected examples. Since the eigenvalue represents the mean squared information captured by its mode, theoretically the LS error can be estimated as [3,4]:

(13) $$\mathrm{Er}r_{\mathrm{LS}}=\sqrt{\sum_{i=M+1}^{N_{s}}\lambda_{i}/\sum_{i=1}^{N_{s}}\lambda_{i}}\;.$$

One can then observe the eigenvalue spectrum in descending order to determine the number of modes (i.e., DoF) needed to reach a desired accuracy. For example, consider a system with the eigenvalue decreasing evidently from one mode to the next and the *M*-th eigenvalue drops three to four orders of magnitude from the first mode. According to Eq. (13), an error near or below 1% is expected from the POD prediction. However, it should be noted that this error prediction is reasonable only if the quality of data collected via DNSs for the POD training is adequate and the solution for $a_{j}$ in Eq. (12) is determined appropriately.

### Example of heat transfer in a CPU

The POD-Galerkin simulation methodology has many applications spanning different fields and problems. One of these applications is the thermal analysis of semiconductor chips [3,4], including central processing units (CPUs) or graphic processing units (GPUs). The high power density in modern semiconductor chips due to their highly integrated devices and interconnects has led to large thermal gradients and high-temperature hot spots that tend to degrade chip performance and damage devices and interconnects. Effective thermal management is thus needed to minimize thermal gradients and hot-spot formation, which however requires accurate, efficient thermal simulations to offer dynamic temperature profiles on the chips.

This example illustrates the implementation of the POD-Galerkin simulation methodology to a heat transfer problem in a CPU. The heat transfer equation is given as:

(14) $$\frac{\partial \rho \mathrm{CT}\left(\vec{r},t\right)}{\partial t}-\nabla \cdot \left(k\nabla T\left(\vec{r},t\right)\right)=P_{d}\left(\vec{r},t\right),$$

where ρ is the density, C is the specific heat, k is the thermal conductivity, and $P_{d}$ is the power density. To derive a set of dynamic equations for $a_{j}$ in Eq. (12), the Galerkin projection of Eq. (14) onto the POD space is performed:

(15) $$\int_{\Omega }\left(\eta \frac{\partial \rho \mathrm{CT}}{\partial t}+\nabla \eta \cdot k\nabla T\right)d\Omega =\int_{\Omega }\eta P_{d}d\Omega +\int_{S}\eta k\nabla T\cdot d\vec{S},$$

where $\vec{S}$ represents the surface vector on the boundary of Ω. Applying Eq. (12) to Eq. (15) with $Q(\vec{r},t)$ replaced by $T(\vec{r},t)$, a set of *M*-dimensional ordinary differential equations (ODEs) can be derived [3,4]:

(16) $$\sum_{j=1}^{M}c_{i,j}\frac{da_{j}\left(t\right)}{\mathrm{dt}}+\sum_{j=1}^{M}g_{i,j}a_{j}\left(t\right)=P_{\mathrm{pod},i},\;\text{for}\;i=1\;\text{to}\;M,$$

where $c_{i,j}$ and $g_{i,j}$ are the elements of thermal capacitance and conductance matrices in the POD space, respectively,

(17) $$c_{i,j}=\int_{\Omega }\rho C\eta_{i}\eta_{j}d\Omega ,\;\text{and}\;g_{i,j}=\int_{\Omega }k\nabla \eta_{i}\cdot \nabla \eta_{j}d\Omega ,$$

and $P_{\mathrm{pod},i}$ includes the projected power density in Ω and heat flux across the boundary surface S onto the POD space,

(18) $$P_{\mathrm{pod},i}=\int_{\Omega }\eta_{i}P_{d}\left(\vec{r},t\right)d\Omega +\int_{S}\eta_{i}k\nabla T\cdot d\vec{S}.$$

Once the POD modes for the CPU are generated, the coefficients in POD space given in Eqs. (17) and (18) can be pre-evaluated and stored in a database for thermal simulation of the selected CPU subjected to heat excitations and BCs.

**Fig. 1:**
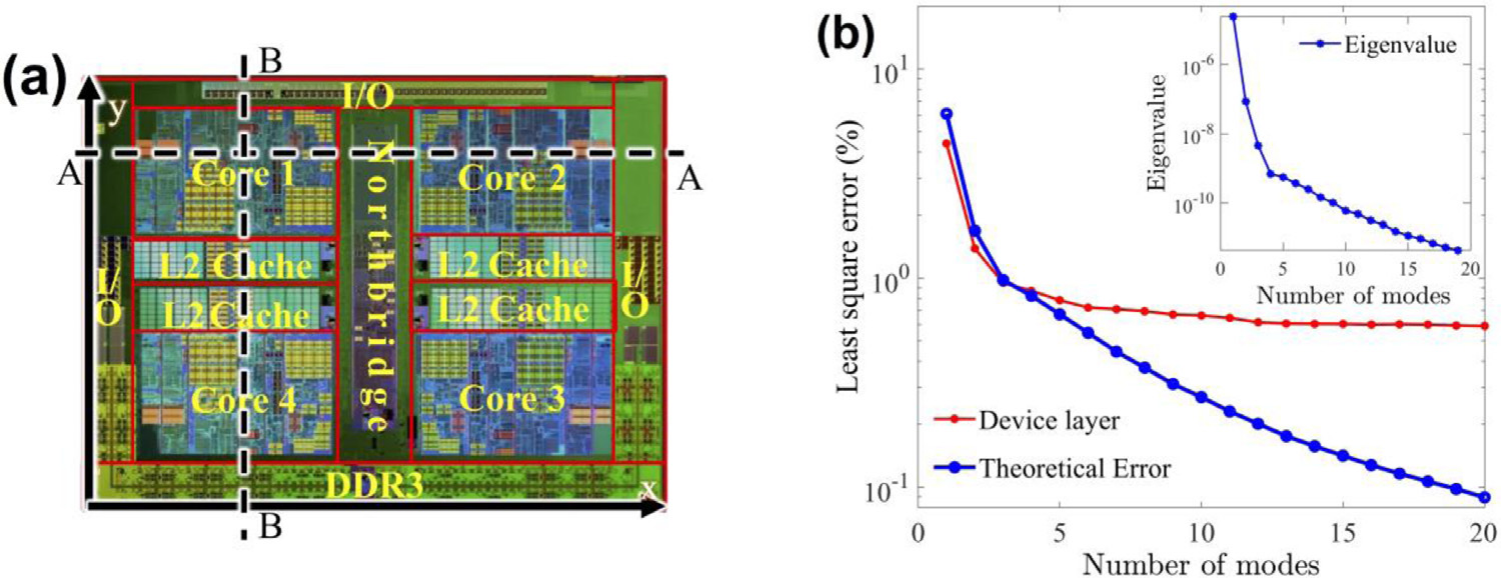
(a) Layout of the AMD ATHLON II CPU [4]. (b) Least square (LS) error of the POD simulation, where the eigenvalue is included in the inset in descending order.

The quad-core AMD ATHLON II X4 610e CPU given in Fig. 1a [4] is selected in this example. The thermal simulation data of the CPU for the POD mode training are obtained from FEM simulation performed in FEniCS [21]. Using the method of snapshots [19,20], POD modes and eigenvalues are generated from thermal data collected from the FEniCS-FEM simulation of the CPU subjected to a dynamic power map. The eigenvalue spectrum is shown in the inset of Fig. 1b, where the eigenvalue decreases by nearly 4 orders of magnitude from the first mode to the third mode. The theoretical LS error given in (13) thus predicts an error near 1% with 3 modes, as shown in Fig. 1b. Beyond 5 modes, the numerical LS error starts deviating from the theoretical prediction and becomes nearly invariant due to the numerical accuracy, which is discussed below together with more detailed thermal profiles predicted by the POD-Galerkin methodology.

**Fig. 2:**
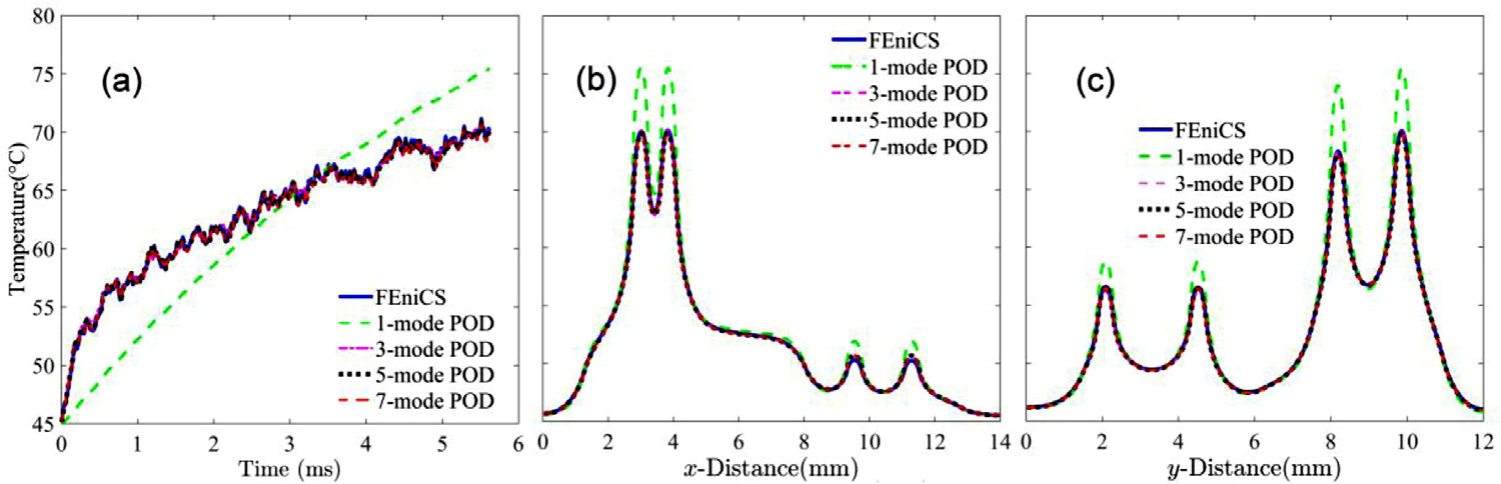
(a) Dynamic temperature at the intersection of Lines A and B, and temperature distributions along (b) Line A and (c) Line B at 6.2 ms. Locations of Lines A and B are indicted in Fig. 1.

The dynamic thermal solution on the device layer predicted by the POD approach subjected to a different dynamic power map is illustrated in Fig. 2, compared with the result obtained from FEniCS-FEM. As shown in Fig. 2a–c, the POD thermal simulation offers exceptionally accurate thermal simulation in time and in space with just 3 modes. Fig. 1b shows that the LS error predicted by the POD simulation with 3 modes is near 1% error. Such a small number of DoF needed in the POD-Galerkin methodology to offer an accurate prediction of the dynamic temperature profile is twofold. First, the effective decomposition procedure given in (1) warrants that the trained POD modes extract essential information embedded in the collected data. Second, the Galerkin projection executed in (15) incorporates the physical principles of heat transfer into the finalized model in (16) to correctly capture the dynamic heat transfer behavior. Although the theoretical LS error estimated by Eq. (13) continues decreasing with the number of modes, the numerical LS error for the POD simulation becomes nearly flattened beyond 5 modes and stays near 0.6% when 12 or more modes are used due to the numerical accuracy of the data used in the training. An LS error below 0.6% can be achieved if the data collection from FEniCS-FEM simulation is performed using a smaller mesh, which will however drastically increase computational times for data collection in FEniCS-FEM simulation, POD mode generation using the method of snapshots in (10) and (11), and calculations of coefficients in (16). For this 3D dynamic heat transfer example, the POD-Galerkin methodology with 3 or 4 modes offers a reduction in DoF by nearly 5 orders of magnitude and an increase in computational speed by 4 orders for predicting the dynamic thermal profile on the device layer.

### Example of quantum nanostructure

This example illustrates the application of the POD simulation methodology to the Schrödinger equation for a quantum nanostructure subjected to external electric filed. The wave function (WF) $\psi (\vec{r})$ of an electron is governed by the Schrödinger equation:

(19) $$\nabla \cdot \left[-\frac{\hbar^{2}}{2m^{*}}\nabla +U\left(\vec{r}\right)\right]\psi \left(\vec{r}\right)=E\psi \left(\vec{r}\right),$$

where ℏis the reduced Plank constant, $m^{*}\;$is the effective mass, $U(\vec{r})\;$is the potential energy, and Eis the total energy of the electron. Different from the heat transfer problem where only one numerical solution is sampled at each snapshot in the training, the data collected from the quantum eigenvalue problem at each snapshot (i.e., at one specific external electric field in this case) include $N_{Q}$ WF solutions for the first $N_{Q}$ quantum states, where $N_{Q}$ is the selected number of quantum states of interest. That is, WFs in all the selected quantum states are trained together to generate only one set of POD modes and eigenvalues for the POD space of all the selected quantum states of the quantum problem. In order to offer enough variations in the training data, the number of snapshots $N_{S}$ must be large enough; i.e., $N_{S}\geq M\geq N_{Q}$.

To incorporate the physical principles in the POD simulation model, Eq. (19) is projected onto the generated POD modes using the Galerkin projection method. This leads to the weak form of Eq. (19) [4,7,8]:

(20) $$\int_{\Omega }\nabla \eta_{i}\cdot \frac{\hbar^{2}}{2m^{*}}\nabla \psi d\Omega +\int_{\Omega }\eta_{i}U\psi d\Omega -\int_{S}\eta_{i}\frac{\hbar^{2}}{2m^{*}}\nabla \psi \cdot d\vec{S}=E\int_{\Omega }\eta_{i}\psi d\Omega \;.$$

After plugging Eq. (12) into Eq. (20) with $Q(\vec{r},t)$ replaced by $\psi (\vec{r})$, the following M×Mquantum eigenvalue problem for $\vec{a}$ in the POD space can be derived as:

(21) $$H_{\eta }\vec{a}=E\vec{a}\;\text{with}\;\vec{a}={\left[a_{1},\;a_{2},\;\ldots ,\;a_{M}\right]}^{T},$$

where the Hamiltonian matrix $H_{\eta }\;$in the POD space is given by [8]:

(22) $$H_{\eta }=T_{\eta }+U_{\eta }+B_{\eta }$$

with the interior kinetic energy matrix $T_{\eta }$ is expressed by:

(23) $$T_{\eta i,j}=\int_{\Omega }\nabla \eta_{i}\left(\vec{r}\right)\cdot \frac{\hbar^{2}}{2m^{*}}\nabla \eta_{j}\left(\vec{r}\right)d\Omega ,$$

the potential energy matrix $U_{\eta }\;$is given by:

(24) $$U_{\eta i,j}=\int_{\Omega }\eta_{i}\left(\vec{r}\right)U\left(\vec{r}\right)\eta_{j}\left(\vec{r}\right)d\Omega ,$$

and the boundary kinetic energy matrix $B_{\eta }$ is of the form:

(25) $$B_{\eta i,j}=-\int_{S}\eta_{i}\left(\vec{r}\right)\frac{\hbar^{2}}{2m^{*}}\nabla \eta_{j}\left(\vec{r}\right)\cdot d\vec{S}.$$

In this example, homogeneous Neumann and Dirichlet BCs are assumed and thus $B_{\eta i,j}=0$.

Solving the quantum eigenvalue problem for $\vec{a}$ in the POD space given by Eq. (21), the WF of the *i* th eigenstate can be reconstructed as:

(26) $$\psi_{i}\left(\vec{r}\right)={\vec{a}}_{i}^{T}\vec{\eta }\left(\vec{r}\right)=\sum_{j=1}^{M}a_{j,i}\eta_{j}\left(\vec{r}\right)\;\text{for}\;1\leq i\leq N_{Q}.$$

**Fig. 3:**
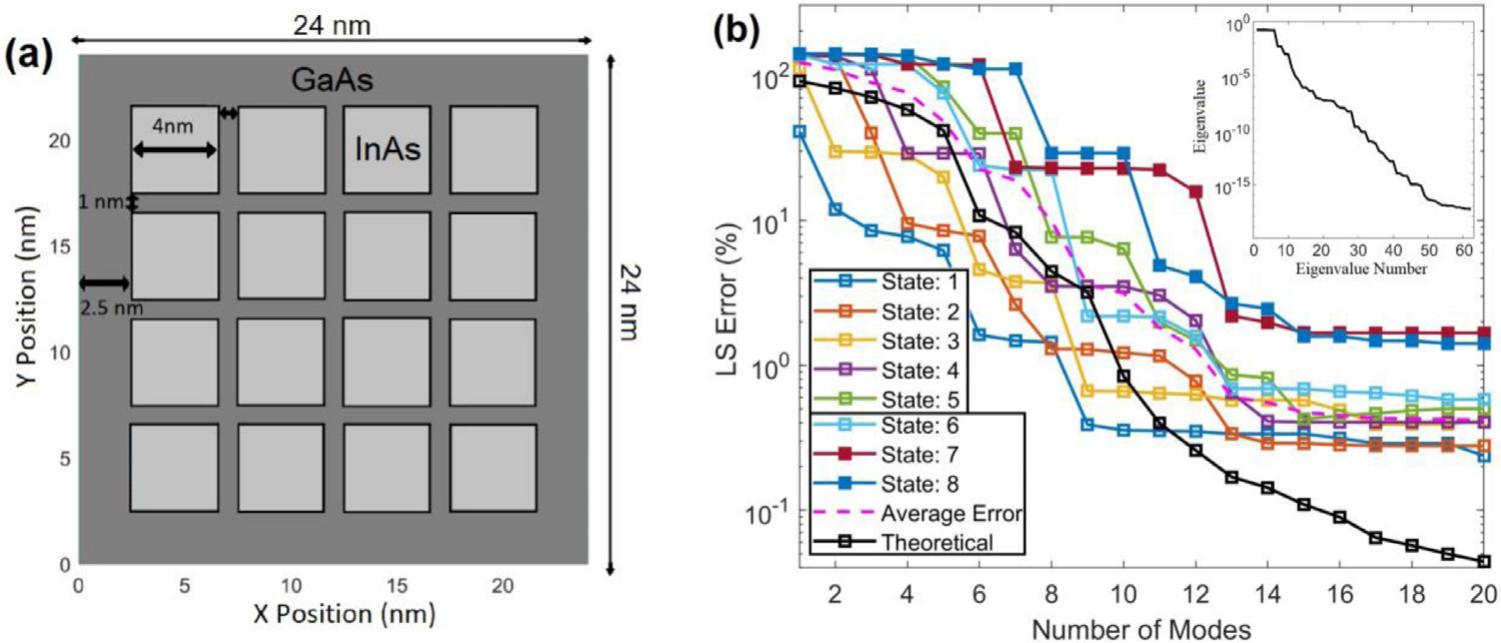
(a) 2D GaAs/InAs quantum dots. (b) POD LS error of WFs in Quantum States 1–8, where States 7 and 8 are not included in the training. The dashed pink line represents the average of the POD LS errors for the trained 6 quantum states while the black line represents the theoretical LS error. The inset is the eigenvalue spectrum for the WF data of the first 6 quantum states.

**Fig. 4:**
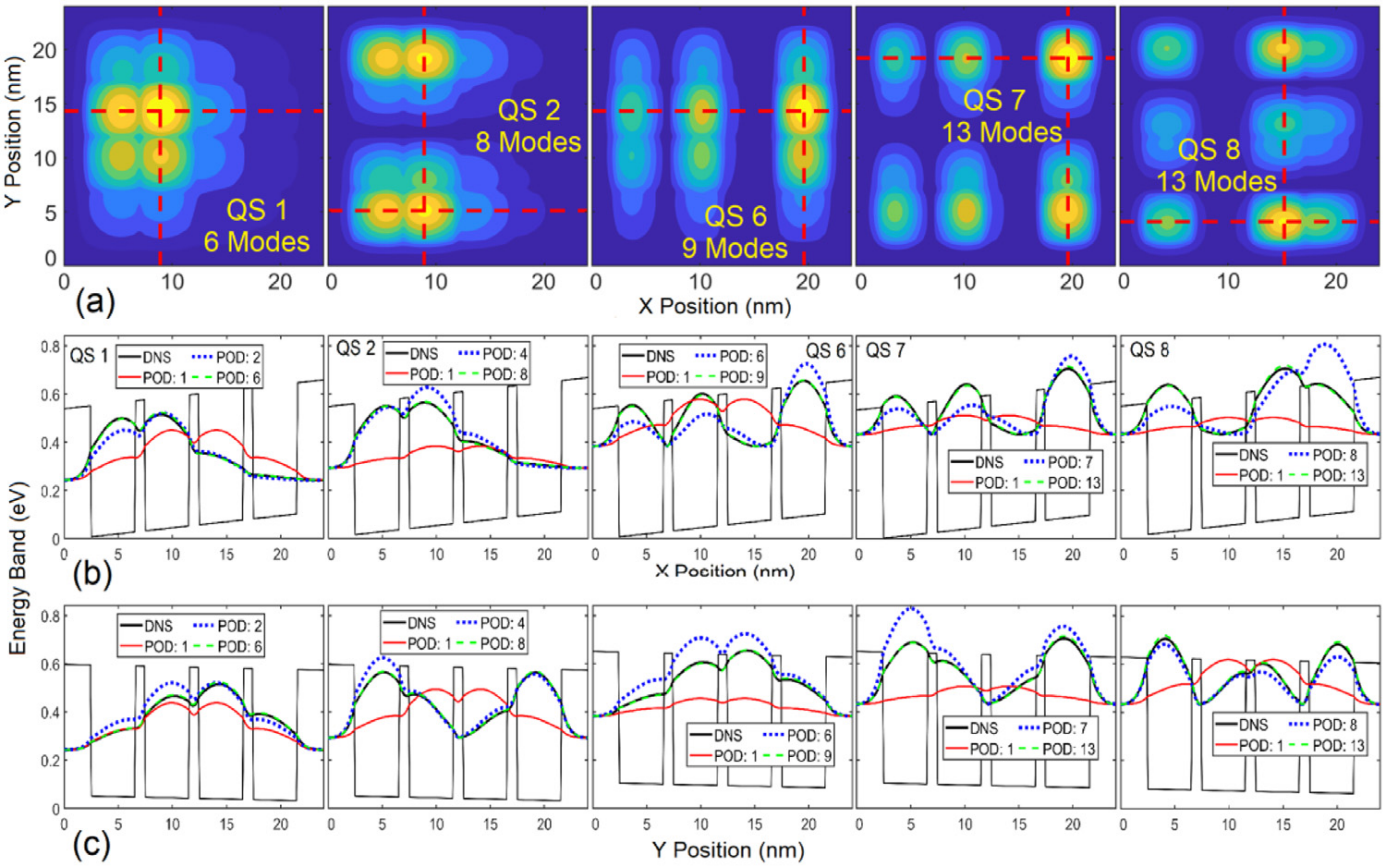
(a) Contours of the electron probability density ${|\psi (\vec{r})|}^{2}$ in several states from the quantum POS simulation model, where the red dashed lines indicate the x and y plotting paths for ${|\psi (\vec{r})|}^{2}$ given below. Profiles of ${|\psi (\vec{r})|}^{2}$ along (b) the x direction and (c) the y direction obtained from both the POD and DNS approaches.

Note that the data collected from DNSs in this example include WFs of the first $N_{Q}$ selected quantum states, and thus, only the first $N_{Q}$ states are trained. However, the M×M POD Hamiltonian equation in Eq. (21) provides M quantum-state solutions ($M\geq N_{Q}$); namely the POD quantum model could possibly offer WFs beyond the trained quantum states, i.e., $i>N_{Q}$ in Eq. (26), if $M>N_{Q}$. Although theoretically the POD WF solutions are only accurate for the trained quantum states, the LS error of the POD WFs for the untrained states with i slightly greater than $N_{Q}$ is in fact reasonably small, as will be seen in Figs. 3 and 4.

The selected nanostructure for this example is a 4 × 4 grid of 2D GaAs/InAs quantum dots (QDs) shown in Fig. 4a. WF data in the first 6 quantum states are collected from DNS of the Schrödinger equation using a FDM, subjected to different applied electric fields. Eight single electric fields, varying from −35 kV/cm to +35 kV/cm, are applied in the two orthogonal x andy directions separately. Together with the sample at zero electric field, there is a total of 17 samples of WFs. Because WFs at the first six Quantum States are collected for each applied electric field, there are 102 snapshots of WFs used in the training. The method of snapshots is applied to generate the POD modes and eigenvalues. It can be noticed in the inset of Fig, 3(b) that the eigenvalue drops by more than 3 orders of magnitude from the first to the 11th mode, and Fig. 3(b) shows that the theoretical LS error estimated from Eq. (13) is below 1% with 10 or more modes. Differently from the heat transfer problem that involves only one solution, the POD modes in this case represent the solutions for WFs in all 6 selected quantum states. Therefore, Eq. (13) offers a theoretical estimate of the LS error averaged over WFs in all 6 trained states [4].

In this example, an electric field of $50\;\hat{x}-10\;\hat{y}$ (kV/cm) is applied to the QD structure to validate the quantum POD-Galerkin methodology, where the *x*-component is higher than the maximum field (35 kV/cm) used in the training. Despite the test condition beyond the training field, the LS error from the quantum POD model is below 0.9% with 13 or more modes for all trained states, as shown in Fig. 3(b). In general, the POD model is more effective in the lower quantum states. As can been seen, the LS error in States 1–3 is near or below 1.2% when 9 modes are included. In States 4- 6, 13 modes are needed to bring the LS error below 2%. When using 15 or more modes, the LS errors in all trained states are below 0.7%. The average LS error given by the pink dashed line more or less follows the theoretical LS error until 10 or 11 modes. In the untrained 7 and 8 states, the LS errors are near 2.1% and 2.6% with 13 modes and stay near 1.7% and 1.4% with more modes, respectively.

Fig. 4(a) shows the contours of the electron probability densities ${|\psi (\vec{r})|}^{2}$ in States 1, 2, 6, 7 and 8 in the QD structure with plotting paths for the profiles of ${|\psi (\vec{r})|}^{2}$ given in Figs. 4(b) and 4(c). The profiles shown in Figs. 4(b) and 4(c) illustrate a very good agreement between the results obtained from the DNS and POD approaches with a handful of POD modes. Fig. 4 further illustrates the accurate WFs predicted by the POD model in all the trained and untrained quantum states influenced by an electric field beyond the training condition. This example has demonstrated the remarkable learning ability of the quantum POD-Galerkin simulation methodology. For this 2D quantum eigenvalue problem, the POD-Galerkin methodology offers accurate solution with a reduction in DoF and computational time by more than three and two orders of magnitude, respectively, even for the untrained states beyond the training fields.

## Conclusion

Concepts, formulation and implementation of the POD-Galerkin simulation methodology have been presented for simulations of physical problems governed by PDEs. The physical problem of interest is projected onto a functional space spanned by an optimal set of POD modes that are trained by good-quality data collected from DNSs. To account for the physical principles, Galerkin projection of the governing equation for the problem is performed, which leads to a finite set of ODEs. High accuracy achieved by this physics-driven learning methodology using a very small number of DoF has been demonstrated against DNSs for two very different physics simulation problems, including a 3D heat transfer problem in a CPU and a 2D quantum eigenvalue problem in a nanostructure. Such an efficient, accurate prediction stems from the fact that the POD learning procedure given in (1) effectively extracts essential information embedded in the collected data and that the Galerkin projection successfully enforces the physical principles in the finalized POD-Galerkin model in (16) or (21). This physics-drive approach also exhibits the extrapolation capability with good accuracy as illustrated in Figs. 3 and 4, where the electric field is beyond the training field and QSs 7and 8 are not included in the training. One of the major advantages is the reasonable error prediction given in (13), which allows users to select the DoF in the POD-Galerkin simulation to reach a desired accuracy.

It has been demonstrated that the methodology offers reductions in the DoF by nearly five and more than three orders of magnitude for the 3D dynamic heat transfer and 2D quantum problems, respectively, compared to DNSs. These increase computational speeds by four and two orders of magnitude for the 3D dynamic heat transfer and 2D quantum problems, respectively. This investigation has suggested possible applications of the rigorous POD-Galerkin methodology to physics simulations spanning many other fields whose physical quantities are described by PDEs.

## CRediT authorship contribution statement

**Alessandro Pulimeno:** Writing – review & editing. **Graham Coates-Farley:** Writing – review & editing. **Martin Veresko:** Software. **Lin Jiang:** Software. **Ming-Cheng Cheng:** Conceptualization, Writing – review & editing. **Yu Liu:** Conceptualization, Writing – review & editing. **Daqing Hou:** Conceptualization, Writing – review & editing.

## Declaration of Competing Interest

The authors declare the following financial interests/personal relationships which may be considered as potential competing interests.

## Data Availability

Code has been published on github. The link to the github page is in manuscript.
